# Supplementary figures and images for: Peptidoglycan recycling is critical for cell division, cell wall integrity, and β-lactam resistance in Caulobacter crescentus
Source: eLife. 2026 Apr 2;14:RP109465. doi: 10.7554/eLife.109465 (PMC13046382; doi:10.7554/eLife.109465)

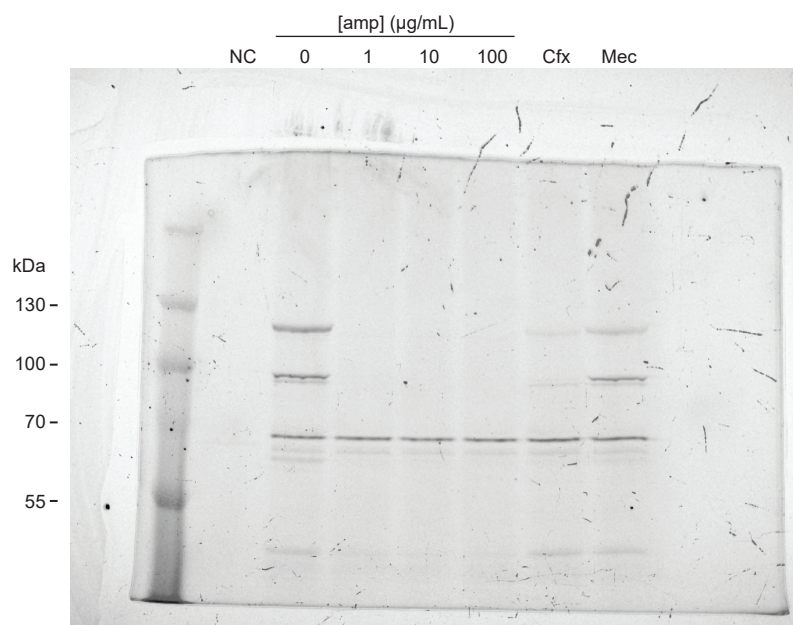

Supplement: Figure 9—source data 1. [file elife-109465-fig9-data1.zip › Figure 9D-source data.pdf]

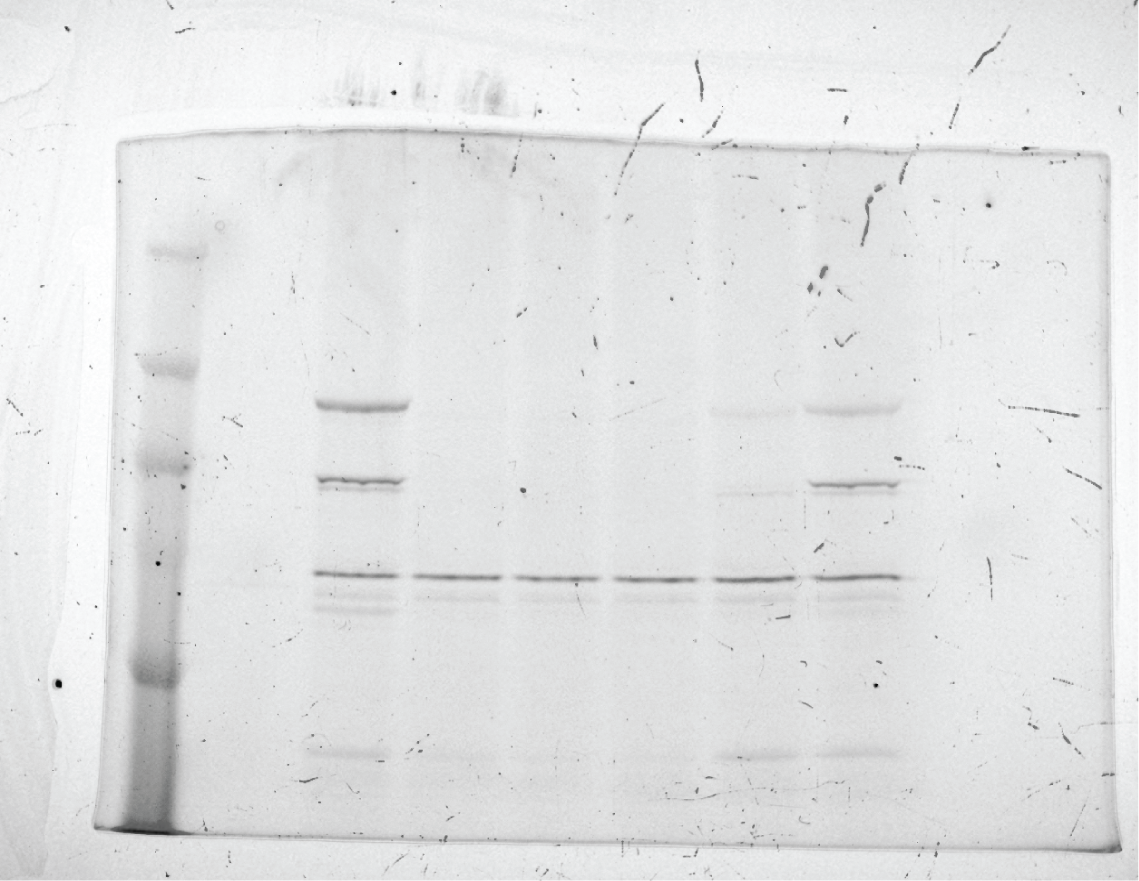

Supplement: Figure 9—source data 2. [file elife-109465-fig9-data2.zip › Figure 9D-source data.tif]
