# Supplementary material for: Peptidoglycan recycling is critical for cell division, cell wall integrity, and β-lactam resistance in Caulobacter crescentus
Source: eLife. 2026 Apr 2;14:RP109465. doi: 10.7554/eLife.109465 (PMC13046382; doi:10.7554/eLife.109465)
Supplement: Supplementary file 1. — The table lists the ORF numbers and predicted functions of the numbered proteins in Figure 8—figure supplement 1C. [file elife-109465-supp1.docx]

**Supplementary file 1. Proteins found to be differentially accumulated in ΔregX cells compared to wild-type cells.** The table lists the ORF numbers and predicted functions of the numbered proteins in **Figure 8–figure supple­ment 1C**.

| **Protein number** | **ORF number** | **Predicted function** |
| --- | --- | --- |
| 1 | *CCNA_00845* | Antitoxin protein RelB-1 |
| 2 | *CCNA_02277* | TonB-dependent outer membrane channel HutA |
| 3 | *CCNA_00081* | uncharacterized protein |
| 4 | *CCNA_03558* | ATP synthase epsilon chain AtpC |
| 5 | *CCNA_02275* | ABC transporter, periplasmic component |
| 6 | *CCNA_00437* | Methyl-accepting chemotaxis protein |
| 7 | *CCNA_03263* | TonB-dependent receptor |
| 8 | *CCNA_01707* | Gluconate 2-dehydrogenase subunit 3-family protein |
| 9 | *CCNA_03446* | Feruloyl-CoA synthetase |
| 10 | *CCNA_01186* | uncharacterized protein |
| 11 | *CCNA_00987* | Stress response protein CsbD |
| 12 | *CCNA_00928* | GntR family transcriptional regulator |
| 13 | *CCNA_00374* | ATP synthase protein I |
| 14 | *CCNA_01205* | ABC transporter ATP-binding protein |
