# Supplementary material for: Peptidoglycan recycling is critical for cell division, cell wall integrity, and β-lactam resistance in Caulobacter crescentus
Source: eLife. 2026 Apr 2;14:RP109465. doi: 10.7554/eLife.109465 (PMC13046382; doi:10.7554/eLife.109465)
Supplement: Supplementary file 2. — The table gives the relative abundance of the indicated muro­pep­tide species, calculated from the areas of the corresponding peaks in the HPLC chromatograms from Figure 9—figure supplement 1. [file elife-109465-supp2.docx]

**Supplementary file 2. Muropeptide composition of peptidoglycan isolated from stationary C. crescentus wild-type and ΔamiR cells.** The table gives the relative abundance of the indicated muro­pep­tide species, calculated from the areas of the corresponding peaks in the HPLC chromatograms from **Figure 9–figure supplement 1**. Two independent replicates were analyzed per strain.

| **Peak no.** | **Muropeptide** | **Peak area (%)** | | | |
| --- | --- | --- | --- | --- | --- |
|  |  | **Wild type** | | **Δ*amiR*** | |
| 1 | Tri | 0.0 | 0.0 | 0.3 | 0.0 |
| 2 | Tetra | 20.2 | 20.1 | 19.2 | 19.3 |
| 3 | Penta(Gly5) | 11.7 | 11.6 | 9.7 | 10.9 |
|  | unknown-possibly Di | 0.6 | 0.7 | 0.4 | 0.7 |
| 4 | Penta | 9.2 | 9.2 | 8.6 | 9.7 |
| 5 | TriTri(Dap) | 0.0 | 0.0 | 0.0 | 0.0 |
| 6 | TetraTri (Dap)) | 0.2 | 0.2 | 1.0 | 0.2 |
| 7 | Tri-Anh (+small amount of TetraTri (D,L)) | 0.7 | 0.7 | 0.3 | 0.7 |
| 8 | TetraTetra(Dap) | 0.0 | 0.2 | 0.2 | 0.2 |
| 9 | TetraPenta(Gly5) | 10.8 | 11.0 | 10.5 | 11.2 |
| 10 | TetraTetra | 13.5 | 13.4 | 16.2 | 13.3 |
| 11 | TetraPenta | 7.6 | 8.2 | 8.1 | 8.9 |
| 12 | TetraTetraTri or TetraTetraTri(Dap) | 0.0 | 0.0 | 0.0 | 0.0 |
| 13 | TetraAnh | 0.5 | 0.0 | 0.2 | 0.0 |
| 14 | TetraTetraPenta(Gly5) | 2.2 | 2.2 | 2.3 | 2.6 |
| 15 | TetraTetraTetra | 2.2 | 2.2 | 4.7 | 2.4 |
| 16 | TetraTetraPenta | 1.1 | 1.2 | 1.2 | 1.3 |
| 17 | TetraTetraTetraTetra | 0.2 | 0.2 | 1.0 | 0.3 |
| 18 | TetraTetraTetraPenta | 0.3 | 0.3 | 0.3 | 0.3 |
| 19 | Penta-Anh | 0.5 | 0.5 | 0.4 | 0.5 |
| 20 | TetraTetra-Anh | 2.4 | 2.2 | 2.4 | 2.3 |
| 21 | TetraPenta-Anh | 2.4 | 2.4 | 2.1 | 2.3 |
| 22 | TetraTetraTriAnh or TetraTetraTri(Dap)Anh | 0.4 | 0.4 | 0.4 | 0.4 |
| 23 | TetraTetraTetra-Anh | 3.4 | 3.3 | 2.7 | 3.0 |
| 24 | TetraTetraPenta-Anh | 2.9 | 2.8 | 2.1 | 2.6 |
| 25 | TetraTetraTetraPenta-Anh I | 0.3 | 0.3 | 0.3 | 0.3 |
| 26 | TetraTetraTetraPenta-Anh II | 0.6 | 0.6 | 0.4 | 0.6 |
| 27 | TetraTetraTetra-diAnh | 1.0 | 1.0 | 0.6 | 0.9 |
| 28 | TetraTetraTetraPenta-diAnh | 0.5 | 0.5 | 0.4 | 0.5 |
| 29 | TetraTetra-diAnh | 0.4 | 0.4 | 0.3 | 0.3 |
| 30 | TetraTetraPenta-diAnh | 0.3 | 0.3 | 0.0 | 0.2 |
