# Supplementary material for: Peptidoglycan recycling is critical for cell division, cell wall integrity, and β-lactam resistance in Caulobacter crescentus
Source: eLife. 2026 Apr 2;14:RP109465. doi: 10.7554/eLife.109465 (PMC13046382; doi:10.7554/eLife.109465)
Supplement: Supplementary file 3. — The table summarizes the relative abundance of different muropeptide species, calculated from the values listed in Supplementary file 2. [file elife-109465-supp3.docx]

**Supplementary file 3. Overview of the muropeptide species identified in peptidoglycan from stationary C. cres­centus wild-type and ΔamiR cells.** The table summarizes the relative abundance of different muro­pep­tide spe­cies, calculated from the values listed in **Supplementary file 2**. Two independent replicates were analyzed per strain.

| **Muropeptide species** | **Peak area (%)** | | | |
| --- | --- | --- | --- | --- |
|  | **Wild type** | | **Δ*amiR*** | |
| All known | 95.8 | 95.9 | 96.1 | 96.0 |
| Monomers (total) | 44.5 | 43.9 | 40.2 | 43.0 |
| Dimers (total) | 38.9 | 39.6 | 42.4 | 40.2 |
| Trimers (total) | 14.0 | 13.9 | 14.5 | 14.1 |
| Tetramers (total) | 1.9 | 2.0 | 2.5 | 2.0 |
| Tripeptides (total) | 0.9 | 1.0 | 1.3 | 1.0 |
| Tetrapeptides (total) | 62.6 | 62.2 | 65.7 | 61.8 |
| Pentapeptides (total) | 35.8 | 36.2 | 32.6 | 36.5 |
| Chain ends (anhydros) | 8.3 | 7.6 | 6.1 | 7.4 |
| Average chain length | 12.0 | 13.1 | 16.2 | 13.4 |
| 3-3 crosslinks | 0.3 | 0.4 | 0.8 | 0.3 |
| Degree of cross-linkage | 30.2 | 30.5 | 32.7 | 31.0 |
| % peptides in cross-links | 55.5 | 56.1 | 59.8 | 57.0 |
