# Supplementary material for: Peptidoglycan recycling is critical for cell division, cell wall integrity, and β-lactam resistance in Caulobacter crescentus
Source: eLife. 2026 Apr 2;14:RP109465. doi: 10.7554/eLife.109465 (PMC13046382; doi:10.7554/eLife.109465)
Supplement: Supplementary file 5. [file elife-109465-supp5.docx]

**Supplementary file 5. Strains used in this study.**

| **Strain** | **Genotype/description** | **Construction** | **Reference/Source** |
| --- | --- | --- | --- |
| ***Caulobacter crescentus*** | | | |
| CB15N | Synchronizable derivative of the wild-type strain CB15 (aka NA1000) | - | [Evinger and Agabian, 1977](#_ENREF_1) |
| AM399 | CB15N Δ*sdpA* | - | [Zielinska et al., 2017](#_ENREF_4) |
| CS606 | CB15N Δ*blaA* | - | [West et al., 2002](#_ENREF_3) |
| ML2103 | CB15N *ftsW::ftsW*_A246T_ | - | [Modell et al., 2014](#_ENREF_2) |
| PR033 | CB15N Δ*amiR* | In-frame deletion of *amiR* in CB15N using pPR029 | This study |
| PR037 | CB15N Δ*amiR* P*_xyl_::*P*_xyl_-amiR* | Transformation of PR033 with pPR037 | This study |
| PR153 | CB15N Δ*traX* | In-frame deletion of *traX* in CB15N using pPR085 | This study |
| PR154 | CB15N Δ*regX* | In-frame deletion of *regX* in CB15N using pPR086 | This study |
| PR173 | CB15N *amiR::amiR*_H26A, H133A, D143A_ | Chromosomal mutation of *amiR* in CB15N using pPR093 | This study |
| PR188 | CB15N Δ*nagZ* | In-frame deletion of *nagZ* in CB15N using pPR099 | This study |
| PR196 | CB15N *nagZ::nagZ*_D259A_ | Chromosomal mutation of *nagZ* in CB15N using pPR101 | This study |
| PR207 | CB15N Δ*ampG* | In-frame deletion of *ampG* in CB15N using pPR109 | This study |
| PR215 | CB15N P*_blaA-O_*-*lacZ* | Transformation of CB15N with pPR110 | This study |
| PR216 | CB15N Δ*amiR* P*_blaA-O_-lacZ* | Transformation of PR033 with pPR110 | This study |
| PR217 | CB15N Δ*nagZ* P*_blaA-O_-lacZ* | Transformation of PR188 with pPR110 | This study |
| PR218 | CB15N Δ*CCNA_02225* | In-frame deletion of *CCNA_02225* in CB15N using pPR111 | This study |
| PR221 | CB15N Δ*amiR* Δ*ampG* | In-frame deletion of *ampG* in PR033 using pPR109 | This study |
| PR246 | CB15N Δ*amiR* *ftsZ::ftsZ*_A246T_ | In-frame deletion of *amiR* in ML2103 using pPR029 | This study |
| PR252 | CB15N Δ*anmK* | In-frame deletion of *anmK* in CB15N using pPR116 | This study |
| PR255 | CB15N Δ*nagK* | In-frame deletion of *nagK* in CB15N using pPR122 | This study |
| PR256 | CB15N Δ*nagA1* | In-frame deletion of *nagA1* in CB15N using pPR123 | This study |
| PR257 | CB15N Δ*nagA2* | In-frame deletion of *nagA2* in CB15N using pPR124 | This study |
| PR258 | CB15N Δ*amiR* Δ*traX* | In-Frame deletion of *amiR* and *traX* in CB15N using pPR127 | This study |
| PR260 | CB15N Δ*amiR* Δ*sdpA* | In-frame deletion of *amiR* in AM399 using pPR029 | This study |
| PR261 | CB15N Δ*amgK* Δ*nagK* | In-frame deletion of *amgK* in PRPR255 using pPR128 | This study |
| PR262 | CB15N Δ*amgK* | In-frame deletion of *amgK* in CB15N using pPR128 | This study |
| PR263 | CB15N Δ*ampG* P*_blaA-O_-lacZ* | Transformation of PR207 with pPR110 | This study |
| PR284 | CB15N Δ*nagZ* P*_xyl_::*P*_xyl_-nagZ* | Transformation of PR188 with pPR137 | This study |
| PR285 | CB15N Δ*ampG* P*_xyl_::*P*_xyl_-ampG* | Transformation of PR207 with pPR119 | This study |
| ***Escherichia coli*** | | | |
| TOP10 | F^–^ mcrA Δ(mrr-hsdRMS-mcrBC) Φ80lacZΔM15 ΔlacX74 recA1 araD139 Δ(ara leu) 7697 galU galK rpsL (Str^R^) endA1 nupG | - | Invitrogen |
| Rosetta(DE3)  pLysS | F^–^ *ompT hsdS*_B_(r_B_^-^ m_B_^-^) *gal dcm* (DE3) pLysSRARE (Cam^R^) | - | Merck Millipore |
| WM3064 | *thrB1004 pro thi rpsL hsdS lacZ*ΔM15 RP4–1360 Δ(*araBAD*)567 Δ*dapA*1341::[*erm pir*(wt)] | - | W. Metcalf (unpublished) |
